# Reported intimate partner violence amongst women attending a public hospital in Botswana

**DOI:** 10.4102/phcfm.v2i1.185

**Published:** 2010-11-04

**Authors:** Lindiwe I. Zungu, Akeem O. Salawu, Gboyega A. Ogunbanjo

**Affiliations:** 1 Department of Health Studies, College of Human Sciences, University of South Africa, South Africa; 2 DRM Hospital, Mochudi, Botswana; 3 Department of Family Medicine & Primary Health Care, University of Limpopo, Medunsa campus, South Africa

**Keywords:** Botswana, intimate partner violence, prevalence, public hospital, women

## Abstract

**Background:**

Intimate partner violence (IPV) is common worldwide and occurs across social, economic, religious and cultural groups. This makes it an important public health issue for health care providers. In South Africa, the problem of violence against women is complex and it has social and public health consequences. The paucity of data on IPV is related to underreporting and a lack of screening of this form of violence in health care settings.

**Objectives:**

The aim of this study was to determine the prevalence of IPV and explore the risk factors associated with this type of violence against women who visited a public hospital in Botswana.

**Method:**

A descriptive, cross-sectional survey was conducted among randomly sampled adult women aged 21 years and older, during their hospital visits in 2007. Data were obtained by means of structured interviews, after obtaining written and signed, informed consent from each participant.

**Results:**

A total of 320 women participated in this study. Almost half (49.7%) reported having had an experience of IPV in one form or another at some point in their lifetime, while 68 (21.2%) reported a recent incident of abuse by their partners in the past year. Experiences of IPV were predominantly reported by women aged 21 – 30 years (122; 38%). Most of the allegedly abused participants were single (173; 54%) and unemployed (140; 44%). Significant associations were found between alcohol use by participants’ male intimate partners (χ^2^ = 17.318; *p* = 0.001) and IPV, as well as cigarette smoking (χ^2^ = 17.318; *p* = 0.001) and IPV.

**Conclusion:**

The prevalence of alleged IPV in Botswana is relatively high (49.7%), especially among young adult women, but the prevalence of reported IPV is low (13.2%). It is essential that women are screened regularly in the country's public and private health care settings for IPV.

## INTRODUCTION

Intimate partner violence (IPV) against women is common worldwide and has been recognised as an important public health problem with immense consequences at both social and political levels.^1^ IPV occurs across all populations, irrespective of social, economic, religious or cultural groups and the only variation is in the patterns of violence in different populations.^2, 3^

There is little consensus among researchers on exactly how to define the term ‘intimate partner violence’, and therefore the definition of the term IPV varies widely from one study to another, making comparisons of the description of the term difficult. IPV is sometimes referred to as domestic violence. The word ‘violence’ is also used interchangeably with ‘abuse’.

In South Africa, the problem of violence against women is complex and has social and public health consequences.^4^ This makes it an important public health issue for health care providers. Most of the time the abuse from IPV is hidden from the public's view and society remains uninformed of the nature and extent of such violence and abuse. Consequently, this form of violence and the extent of the problem cannot be directly observed.

Of concern is the paucity of data on IPV due to ‘underreporting’ and ‘lack of screening’, which contributes to the complexity and the magnitude of this form of violence, especially against women. Furthermore, obtaining reliable data on IPV is a complicated and intricate task, because of the private and intimate context in which this form of violence and abuse often takes place and also because of methodological challenges imposed by the nature of such studies.^5^

Research on violence against women is considered an important objective of any programme designed to eradicate this problem. At the fourth World Conference on Women held in Beijing in 1995, one of the strategic objectives established was to study the causes and consequences of violence against women and the efficacy of preventive measures, thus encouraging governments and organisations to promote research in this area.^6^

A World Health Organization's (WHO's) population-based survey on women's health and domestic violence against women in 2005 established that 10% – 69% of women had been assaulted by their male intimate partners at some stage in their lives, and that 15% – 30% had been assaulted in the previous year.^7^ The objective of this study was to develop methodologies to measure violence against women and its health repercussions in different cultures.

Furthermore, the results of the WHO survey showed that the national lifetime prevalence of partner abuse among South African women was estimated at 13% and occurrence in the previous year at 11%. The Eastern Cape was found to have the highest lifetime prevalence of 27% and previous-year prevalence of 11%.^4^ In Africa, men are seen as the head of the marriage or relationship, which indirectly promotes either verbal or physical abuse towards their female partners.^8^ The latter has also been observed in Mochudi, a village in Botswana, which shares common socio-cultural features with other southern African countries.^9^

## ETHICAL CONSIDERATIONS

Ethical clearance for the study was granted by the Medunsa Research Ethics Committee of the University of Limpopo (MCREC/PH/114/2007), South Africa. The study was conducted following the standard ethical guidelines on conducting gender-based violence studies as stipulated by the WHO,^10^ and the recommendations prescribed in the ethical and safety guidelines for research on domestic violence.^11^ Written and signed informed consent was obtained from each participant prior to data collection. Special arrangements were made for participants to be interviewed after their consultations. Participants were informed of their voluntary participation and that they could decline to participate or withdraw from the study at any time, without any negative consequences. Confidentiality and anonymity of participants were maintained throughout the study by non-disclosure of information obtained and analysis of data as ‘group’ data. Participants who had psychological distress during the interviews were referred to the hospital social worker for counselling and follow-up care, as pre-arranged by the researchers.

## METHODS

This study was conducted to determine the prevalence of IPV and explore the risk factors associated with this type of violence against women who visited a public hospital in Botswana. A descriptive, cross-sectional survey was conducted among adult female participants aged 21 years and older (the lower age limit was chosen because of consent rules in Botswana) who sought medical care for themselves or their children in a public hospital in Botswana. Participants were recruited after their medical history was taken, in order to exclude those with serious physical illnesses (e.g. uncontrolled blood sugar, major injuries, and unconscious, agitated or depressed patients). Moreover, participants were interviewed after their consultation, as their medical conditions could then be considered as stable. A hospital setting was used for this study in order to reduce the risk of further abuse of participants, especially if accompanied by their abusive intimate partners.^12^ The health setting has been identified as one of the best contexts in which IPV can be identified and studied, mainly because of its accessibility and because women who have experienced this form of violence or abuse make greater use of health services than those who have not suffered such an experience.^13, 14^

Two trained, female research assistants fluent in Setswana (participants’ home language) and English assisted with data collection. The statistics program SPSS version 14.0 was used to analyse data and a chi-square test and univariate logistic regression analysis were used to determine the relationships between socio-demographic characteristics and behavioural risk factors associated with IPV. Odds ratios were obtained for each risk factor and logistic regression analysis was done to determine the relationship between the risk factors and IPV.

## RESULTS

From a sample of 352 that was recruited, a total of 320 adult women who attended the outpatient department at a public hospital in Botswana consented to and participated in this study (a 90.9% response rate). Participants were enrolled consecutively over a three-month period on outpatient days that were quieter – hence some days were omitted. Some participants were recruited and agreed to participate in the study but because the interviews were conducted after their consultations, they did not come back to the researchers as they were in a hurry to return home.

### Socio-demographic characteristics

**FIGURE 1:**
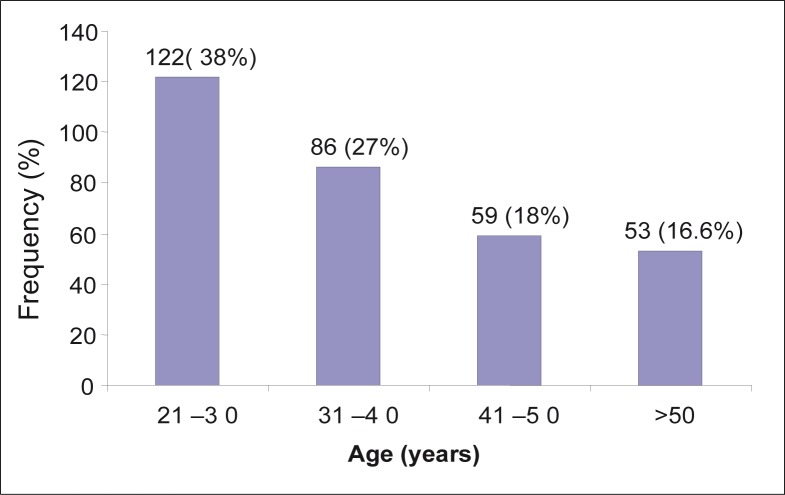
Frequency distribution of the age of the participants in years.

Figure 1 shows the frequency distribution of participants’ age groups in years. The age group 21–30 years comprised 122 (38%) participants and 86 (27%) subjects were in the age group 31–40 years.

**FIGURE 2:**
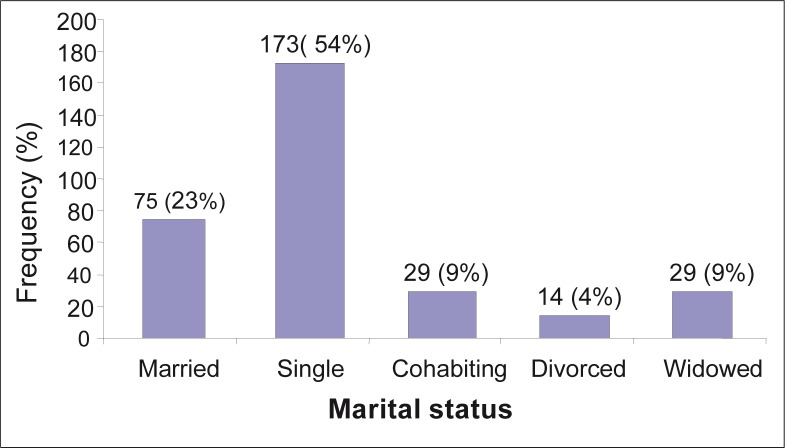
Frequency distribution of the marital status of the participants

Results indicated the participants’ marital statuses at the time of the study as follows: 173 (54%) were single, 75 (23%) were married, 14 (4%) were divorced and equal proportions indicated that 29 (9%) were widowed and cohabiting with their intimate partners (Figure 2).

**FIGURE 3:**
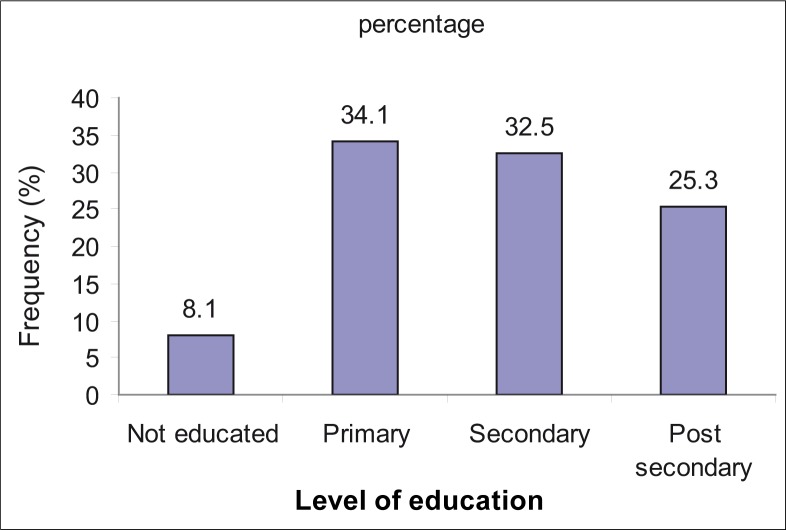
Frequency distribution of participant's highest level of education

The majority (91.9%) indicated that they had formal education, of which 25% had post-secondary level education. Only 8.1% indicated that they did not have any formal education (Figure 3).

### Participants’ employment status and income

According to the results, 140 (44%) participants were unemployed and had no source of income, 117 (37%) were formally employed either by the government or in private establishments, 27 (8%) were self-employed and 11% were engaged in casual jobs. Of those employed, 78 (43%) indicated that they earned below BWP1000 per month (approx. USD140; Table 1).

**TABLE 1:** Employment status and income of participants

Employment status	Income (BWP)†					Total

	No income	< 1000	P1000–2499	P2500–4999	P5000–10 000	
Unemployed	140	0	0	0	0	140
Casually employed	0	29	4	2	1	36
Employed: formal	0	36	44	14	23	117
Self-employed	0	13	11	3	0	27

**Total**	**140**	**78**	**59**	**19**	**24**	**320**

† BWP: Botswana Pula (USD1 = BWP6.75).

### Prevalence of IPV

**FIGURE 4:**
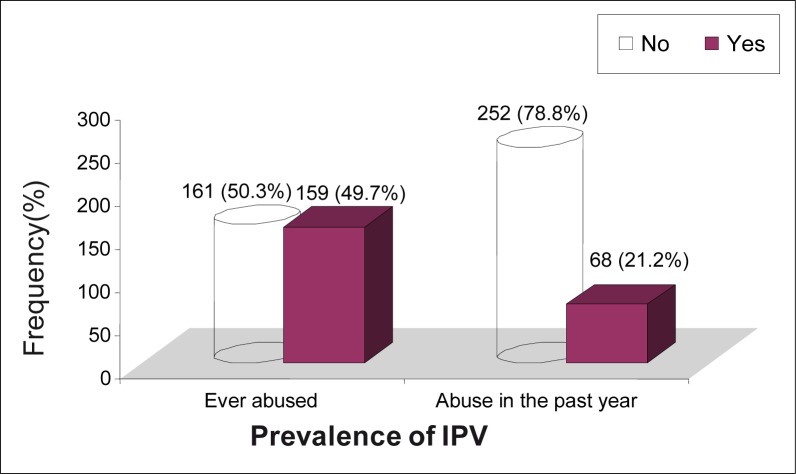
Lifetime and one year prevalence of Intimate Partner Violence among participants

Participants were asked if they had experienced any form of abuse in their relationships with their intimate male partners (i.e. either their current or previous partners, or spouses). This was categorised into: (1) a lifetime, or (2) within-one-year experience of IPV. As depicted in Figure 4, almost half of the participants (159; 49.7%) had experienced IPV in one form in their lifetimes, and 68 (21.2%) had had a recent experience of IPV (i.e. within the previous year).

### Financial dependence on intimate partners

Participants were asked if they were financially dependent on their male partners. Responses indicated that 208 (65%) denied financial dependence, compared to 112 (35%) who admitted to such dependence, as they otherwise had no source of income, being unemployed.

### Substance use by participants’ intimate partners

Slightly more than half of the participants (168; 52.4%) stated that their intimate partners consumed alcohol, of which 54 (16.9%) were habitual alcohol drinkers (Table 2). About two-thirds of the participants (64.1%) declared that their intimate partners did not use any other recreational drugs. A third (106; 33%) of the participants’ intimate partners smoked cigarettes and only a small proportion (9; 2.8%) indicated that their partners smoked dagga (marijuana) and used other illegal drugs.

**TABLE 2:** The use of alcohol and other drugs by intimate partners

	No. of participants	%
**Use of alcohol**		
Never	152	47.5
Habitual	54	16.9
Occasional	114	35.5
**Total**	**320**	**100**
**Use of other drugs**		
None	205	64.1
Cigarettes	106	33.1
Marijuana (dagga)	9	2.8
**Total**	**320**	**100**

### Other factors associated with IPV

Participants were asked about known risk factors associated with IPV. Table 3 shows that the risk factors considered included, (1) infidelity, (2) questioning of male partners about girlfriends, (3) performing activities against the male partner's wish, (4) the influence of alcohol, (5) personality disorders in male partners, (6) asking the male partner for money when he is financially disadvantaged and (7) refusing to have sexual intercourse with him. The odds ratios were greater than 1 for all these risk factors (Table 3). Almost all risk factors except the influence of alcohol and personality disorders in male partners were statistically significant (*p*-value < 0.005). Chi-square analysis showed a significant association between participants’ marital status and lifetime experience of abuse (*c*^2^ = 23.970; *p* < 0.001) and experience of abuse in the previous year (*c*^2^ = 14.743; *p* = 0.005).

**TABLE 3:** Associated risk factors to IPV

Risk factor	Participants		Lifetime		*p–*value
	
	Number	%	IPV	95%CI	
**Infidelity**					
Yes	135	42.2	23.26	9.31–58.09	*p* < 0.0001*
No	185	57.8	-	-	-
**Questioning male partner**					
Yes	90	28.1	9.24	3.04–28.04	*p* < 0.0001*
No	230	71.9	-	-	-
**Activities undertaken against the wish of male partner**					
Yes	70	21.9	63.81	7.10–73.42	*p* < 0.0001*
No	250	78.1	-	-	-
**Alcohol influence**					
Yes	40	12.5	9.55	0.76–119.51	*p* = 0.08
No	280	87.5	-	-	-
**Personality disorder**					
Yes	53	16.6	3.85	0.42–35.51	*p* = 0.234
No	267	83.4	-	-	-
**Requesting money**					
Yes	16	5.0	3.89	1.52–2.48†	*p* = 0.002*
No	304	95.0	-	-	-
**Refusal of sex**					
Yes	35	10.9)	7.26	3.03–5.02†	*p* < 0.0001*
No	285	89.1	-	-	-

* Statistically significant.

† Exact analysis to cater for quasi-separation of data

### Reporting of abuse/help-seeking behaviours

Participants who reported experiences of IPV in their lifetime (159; 49.7%) were asked to indicate the person(s) they had reported the experiences of abuse to. Slightly more than half of the participants (51%) had reported their IPV experiences to close relatives and 40 (25.1%) had reported to the police. Eight respondents (5.03%) stated that they had reported IPV experiences to the nearest hospital and an equal number of participants reported it to other people, mainly social workers (15; 9.4%) and friends (15; 9.4%). The remaining participants (161; 50.3%) indicated that they did not report their IPV experiences for various reasons (not explored in this paper).

## DISCUSSION

### Socio-demographic characteristics

The results of this study showed that IPV is common in young women and those who are economically dependent on their male partners. These findings concur with those from other national surveys done globally, which show that IPV is more common among women than men.^1, 7^ A WHO report on violence and health indicates that young women and those below the poverty line are disproportionately affected by IPV. ^11^ Previous reports on IPV have shown that the young ages of male perpetrators and their victims could also be a risk factor to violence and abuse because of various factors such as immaturity, poverty and unemployment.

### Prevalence of IPV

The results of this study estimate the lifetime and past-year prevalence of IPV to be 49.7% and 21.2%, respectively. This indicates a high prevalence of IPV among the study population, that is, one in every two and one in every five adult female patients who visited the out-patient department of the institution had experienced lifetime and past-year IPV, respectively. These findings are similar to those from a study conducted in China in 2005, which showed the lifetime and past-year prevalence estimations to be 43% and 26%, respectively.^15^ In a similar study conducted in Uganda in 2003, the authors estimated the lifetime prevalence of partner abuse among women to be 54%, while the past-year prevalence was 14%.^16^

In South Africa, the results of a WHO population-based survey carried out in 1998 estimated the national lifetime prevalence of IPV among women to be 13% and the past-year prevalence of IPV to be 11%. Furthermore, provincial figures established that the Eastern Cape province had the highest lifetime prevalence of 27% and a past-year prevalence of 11%.^17^ Findings from a similar study conducted among female patients in a public hospital in Durban (KwaZulu-Natal province) showed an estimated lifetime prevalence of IPV of 38%.^18^ Data from health care-based IPV prevalence studies in the United Kingdom in 2004 estimated lifetime and past-year prevalence to be 12% – 46% and 6% – 28% respectively,^19^ while in the United States of America in 2002, it was 30% – 39% and 6% – 23%, respectively.^20^ Although IPV is regarded as a global concern, the challenge of its underreporting affects the prevalence estimates and magnitude of this form of abuse.^2, 19^

In 1999, a national study on violence against women conducted in Botswana by the Women's Affairs Department found that violence against women was an immense problem, with three out of every five women having been a victim of such violence.9 In a similar study in Uganda, the authors estimated the lifetime prevalence of partner abuse among women to be 54%, while the past-year prevalence was 14%.^16^A London-based study in 2002 on domestic violence revealed that the lifetime prevalence of partner violence in health care settings was 41% and the past-year prevalence was 17%.^20^

### Other risk factors relating to IPV

Several risk factors have been ascribed to IPV, including socio-demographic, cultural and behavioural factors. In this study, as reported by the participants, infidelity and actions against the wishes of the male partners were the most common risk factors for IPV, with odds ratios of 23.26 (95% CI: 9.31–58.09) and 63.81 (95% CI: 7.10–573.42), respectively. Other factors with statistically significant *p*-values included the participants’ questioning of male partners about their extra-marital relationship(s) (*p* < 0.0001), financial dependence on the male partner (*p* = 0.002), and refusing to engage in a sexual relationship (*p* < 0.0001). All were found to increasingly expose participants to IPV.

Regarding alcohol and other substances used by male partners, although there was a significant association with IPV (*c*^2^ = 40.967; *p* < 0.001) it was not reported by most of the participants as contributing to their experience of IPV. The most common behavioural risk factors associated with IPV found in studies carried out in China, USA, UK, South Africa, Uganda and Malawi are (1) extramarital affairs, (2) use of alcohol and other drugs, (3) doing things against the wishes of the male partner and (4) arguments about monetary issues (especially when the male partner is financially disadvantaged).^2, 4, 16, 19, 21^ Results from other studies revealed that alcohol did not initiate IPV, but worsened its outcomes in terms of severity.^19, 22^

### Association between socio-demographic variables of participants and IPV

Several studies have revealed an association between socio-demographic characteristics of both the victims of IPV and the offenders and significant relationships.^1, 3, 14, 15^ The following factors have been identified as factors that increase the risk of women to IPV, (1) a relatively young age, (2) poverty or unemployment, (3) being divorced or separated and (4) a low level of education.^1, 2, 7, 14^

The results of this study further showed a strong relationship between the ages of participants and the occurrence of abuse. This association was only significant (*c*^2^ = 7.991; *p* = 0.035) for a lifetime experience of abuse, where the chances of abuse decreased with increasing age of participants. Such findings (that IPV occurs more frequently in younger people) was also supported by findings from a study conducted in London in 2002, UK, which confirmed that women younger than 45 years (with the highest risk occurring between the ages of 16 and24 years) have a higher risk of experiencing IPV than those older than 45 years.^20^ This has been ascribed to immaturity and lack of patience among partners in a relationship.^1^ Results of this study did not reveal any statistically significant association between age and a past year's experience of abuse (*c*^2^ = 4.131; *p* = 0.248), which is similar to results of a similar study carried out in China.^15^ The reason for this difference is not clear.

This study found a strong association (*c*^2^ = 23.970; *p* < 0.001) between marital status and lifetime experience of abuse, with the highest occurrence among divorced participants (93%), followed by cohabiting partners (75%), singles (49%) and married partners(40%) and the lowest occurrence among widowed participants (34%). This trend was also found with a past-year experience of abuse. A similar trend and order of occurrence, was found in the National Violence Against Women Survey carried out in the USA in 2000.^2^ Several other studies conducted in Botswana, South Africa, USA, China and Malawi also reported similar associations, with the highest occurrence among unmarried and cohabiting couples.^9, 15, 17, 22^

Studies conducted in 2005 in South Africa and Malawi have found that a lower educational status exposes women to IPV.^17, 21^ The results of this study did not find any significant association (*c*^2^ = 4.586; *p* = 0.205) between the educational level of participants and the occurrence of abuse: both educated and uneducated women experienced abuse equally. This was supported by the findings of a Ugandan study, which established that the educational level of women only reduced the frequency of IPV but did not prevent it.^16^

Unemployment, poverty and economic dependence of women expose them to IPV.^1, 2, 7^ This study found that about 44% of all participants were unemployed and therefore did not earn any income, 35% depended economically on their male partners, while others depended on parents and relatives. A significant relationship (*c*^2^ = 14.890; *p* = 0.005) was found between the ‘economic status’ of abused participants and the experience of abuse and the risk decreased as the amount of income that was earned increased. This is in agreement with studies carried out in Africa, Asia and America, which found that women with low socio-economic status are more prone to abuse.^2, 7, 16, 19, 22^

A systematic review of relevant studies conducted in the USA, UK and Malawi found that the low educational levels of women predispose them to abuse by their intimate partners.^2, 19, 21^ A similar trend was found by researchers in a South Africa national survey on partner violence.^18^ A high level of education, however, does not rule out violence: it sometimes predisposes women to abuse, especially if the male partner is less educated.^1, 7, 14^

The consumption of alcohol, a major cause of many social vices, has been found to be related to IPV. A number of studies reveal that alcohol only contributes to violence rather than causing it, and that those women who live with heavy drinkers are at far greater risk of physical partner violence, which tends to be more serious, than that found in those not consuming alcohol.^19, 22^ The results of this study also indicated a very strong association between the frequency of use of alcohol and other drugs (both with *p* < 0.001) by the male partner and the occurrence of IPV in their relationships. There are significant differences between studies in terms of the methods used to measure the presence or absence of alcohol and in the definition of alcohol consumption as a risk factor for the development of violent behaviour. A Chinese study revealed that the use of cigarettes and other drugs increased the chances of a man to abuse his female partner.^15^ This is consistent with the significant association between the use of other drugs (mainly cigarettes) and occurrence of abuse found in this study.

### Economic dependence on male partner and occurrence of abuse

As indicated earlier, studies conducted in the USA, China, Malawi and South Africa found that economic independence of women protected them from various forms of abuse by their male partners.^15, 17, 19, 21^ The results of this study are not consistent with these findings, since no statistically significant association was found between participants’ experiences of abuse and economic dependence or independence on their male partners (*c*^2^ = 0.447; *p* = 0.504). The majority of the women who participated in this study stated that they were not economically dependent on their male partners but, despite such confirmation of financial independence, about half of them still experienced abuse and the overall prevalence of abuse was high. Findings from a study conducted in Botswana reported that almost half of the households in Botswana are headed by women who sustain their families, including their male partners or husbands.^9^ Our results indicated that the majority of the participants were unemployed and earned no income. It did not include consideration of others upon whom the women could depend for financial support.

### Study limitations

This study was limited to one public health institution and, although a high response rate was achieved, the views of other women from other institutions, or in the community, regarding their experiences of IPV, could not be explored. Furthermore, because this study was self-reported and retrospective, the risk of ‘recall’ bias from participants cannot be overruled. In this study participants were recruited in a health care setting, which increases the chance of a ‘selection’ bias, as it is known that women who experienced violence and abuse make greater use of health services, resulting in a possible overestimation of the prevalence of IPV in the general population. On the other hand, because of the methodological limitations imposed by the nature of this study, the researchers had to protect participants from further harm and abuse by their partners, as recommended in the ethical and safety guidelines for research on domestic violence.^13^ For this reason, participants who were accompanied by their partners were not included in the study and thus valuable data could have been overlooked.

## CONCLUSION

IPV is a common public health problem globally. The prevalence of alleged IPV in Botswana is relatively high (49.7%), especially among young adult women, but the prevalence of reported IPV is low (13.2%). It is essential that women are regularly screened in public and private health care settings for undisclosed IPV. In order to quantify the magnitude of such violence, and to implement interventions that can lead to improved outcomes for women who have experienced IPV, it is crucial that clinical guidelines be made available to enable health care professionals to conduct effective screening for IPV. It is our view that the risk of benefit versus harm could be determined once the magnitude of IPV has been established through IPV screening.
